# CRISPR/Cas9 editing of the polygalacturonase *FaPG1* gene improves strawberry fruit firmness

**DOI:** 10.1093/hr/uhad011

**Published:** 2023-02-01

**Authors:** Gloria López-Casado, Cristina Sánchez-Raya, Pablo D Ric-Varas, Candelas Paniagua, Rosario Blanco-Portales, Juan Muñoz-Blanco, Sara Pose, Antonio J Matas, Jose A Mercado

**Affiliations:** Departamento de Botánica y Fisiología Vegetal, Instituto de Hortofruticultura Subtropical y Mediterránea ‘La Mayora’ (IHSM-UMA-CSIC), Universidad de Málaga, 29071 Málaga, Spain; Departamento de Botánica y Fisiología Vegetal, Instituto de Hortofruticultura Subtropical y Mediterránea ‘La Mayora’ (IHSM-UMA-CSIC), Universidad de Málaga, 29071 Málaga, Spain; Departamento de Botánica y Fisiología Vegetal, Instituto de Hortofruticultura Subtropical y Mediterránea ‘La Mayora’ (IHSM-UMA-CSIC), Universidad de Málaga, 29071 Málaga, Spain; Departamento de Botánica y Fisiología Vegetal, Instituto de Hortofruticultura Subtropical y Mediterránea ‘La Mayora’ (IHSM-UMA-CSIC), Universidad de Málaga, 29071 Málaga, Spain; Departamento de Bioquímica y Biología Molecular, Universidad de Córdoba, 14071, Córdoba, Spain; Departamento de Bioquímica y Biología Molecular, Universidad de Córdoba, 14071, Córdoba, Spain; Departamento de Botánica y Fisiología Vegetal, Instituto de Hortofruticultura Subtropical y Mediterránea ‘La Mayora’ (IHSM-UMA-CSIC), Universidad de Málaga, 29071 Málaga, Spain; Departamento de Botánica y Fisiología Vegetal, Instituto de Hortofruticultura Subtropical y Mediterránea ‘La Mayora’ (IHSM-UMA-CSIC), Universidad de Málaga, 29071 Málaga, Spain; Departamento de Botánica y Fisiología Vegetal, Instituto de Hortofruticultura Subtropical y Mediterránea ‘La Mayora’ (IHSM-UMA-CSIC), Universidad de Málaga, 29071 Málaga, Spain

## Abstract

Firmness is one of the most important fruit quality traits in strawberries. The postharvest shelf life of this soft fruit is highly limited by the loss of firmness, where cell wall disassembly plays an important role. Previous studies demonstrated that the polygalacturonase FaPG1 has a key role in remodelling pectins during strawberry softening. In this study, *FaPG1* knockout strawberry plants have been generated using the CRISPR/Cas9 system delivered via *Agrobacterium tumefaciens*. Ten independent lines, cv. “Chandler”, were obtained, and all of them were successfully edited as determined by PCR amplification and T7 endonuclease assay. The targeted mutagenesis insertion and deletion rates were analyzed using targeted deep sequencing. The percentage of edited sequences varied from 47% up to almost 100%, being higher than 95% for seven of the selected lines. Phenotypic analyses showed that 7 out of the eight lines analyzed produced fruits significantly firmer than the control, ranging from 33 to 70% increase in firmness. There was a positive relationship between the degree of *FaPG1* editing and the rise in fruit firmness. Minor changes were observed in other fruit quality traits, such as colour, soluble solids, titratable acidity or anthocyanin content. Edited fruits showed a reduced softening rate during postharvest, displayed a reduced transpirational water loss, and were less damaged by *Botrytis cinerea* inoculation. The analysis of four potential off-target sites revealed no mutation events. In conclusion, editing the *FaPG1* gene using the CRISPR/Cas9 system is an efficient method for improving strawberry fruit firmness and shelf life.

## Introduction

The cultivated strawberry (*Fragaria* × *ananassa*, Duch.) is one of the most popular species in the *Rosaceae* family. The organoleptic properties of strawberries make this fruit one of the most economically important crops cultivated and consumed worldwide. Additionally, this fruit is relevant for its nutritional properties and beneficial effects on health [1]. The soft texture of strawberries determines their short postharvest life and causes significant economic losses. In strawberry breeding programs, improving flesh firmness and maintaining high fruit quality standards is a common goal [2], but the achievements have been limited.

The disassembly of the cell wall and the dissolution of the middle lamella are the primary causes of fruit softening [3, 4]. Cell wall enzymes and proteins related to this process act over the main components of the primary cell walls, decreasing wall strength and cell to cell adhesion [5, 6]. Pectin is the cell wall component that undergoes the most extensive changes during strawberry softening. In general, the concentration of soluble pectins, loosely bound to the wall, increases as the fruit ripens at the expense of ionically- and covalently-bound pectins [7–10]. The length and nanostructural complexity of bound pectins also decrease significantly [10]. Functional analyses have demonstrated that pectinase enzymes, such as polygalacturonases (PGs), pectate lyases, and β-galactosidases, play critical roles in fleshy fruit softening. Transgenic silencing of some of these ripening-specific genes reduced softening and increased postharvest shelf life in fruits with different textural characteristics, such as strawberries and apples [11–13]. PGs are the most studied pectinases involved in fruit softening [14].

Two different PG genes have been described in strawberry, *FaPG1* (accession no. AF380299) and *FaPG2* (accession no. AY280662) [14–16]. They both are up-regulated during fruit ripening [17]. In the case of *FaPG1*, silencing by antisense transformation significantly increased fruit firmness, while no other ripening-related traits such as colour, weight, or soluble solids were affected [14].

Traditional breeding can be a slow process for introgressing a desirable trait into a particular genotype. Difficulty increases in crops with high ploidy levels such as the octoploid *F.* × *ananassa* [18]. In the last decade, a new technology for plant genetic improvement has been developed to precisely edit genes through CRISPR for traits of interest [19]. Successful genome editing has been reported in many crop species, such as sorghum, rice [20, 21], tomato [22, 23], apple [24, 25] and the diploid wild strawberry *Fragaria vesca* [26–28]. Few attempts to modify agronomic traits by gene editing have been described in the cultivated strawberry. CRISPR/Cas9 has been used for editing the endogenous marker gene phytoene desaturase [18], the MADS-box TM6 involved in flower development [29] and the *RAP* gene encoding a glutathione S-transferase that binds anthocyanins [30]. This study aimed to generate, for the first time, edited strawberry plants with improved fruit quality traits. The CRISPR/Cas9 system was successfully used for editing the endopolygalacturonase *FaPG1* in cv. “Chandler”, a soft-fruit genotype with excellent eating quality [31].

## Results

### Transformation and regeneration of *FaPG1* edited plants

The genomic sequence of *FaPG1* (accession no. AF380299) was previously identified in cv. “Chandler” [15]. This sequence was searched in the most recent *Fragaria* × *ananassa* genomes (https://www.rosaceae.org/). *FaPG1* was annotated as FxaC_21g15770 (Camarosa genome v1.0.a2, [32]) or Fxa6Ag103973 (Royal Royce genome v1.0, [33]), both located in chromosome 6A, which displayed 99% identity values with the query sequence. The sequence FxaC_21g15770 is 2953 bp long and contains four exons, encoding for a PG protein of 405 aas. However, the sequence obtained in the Royal Royce reference genome (Fxa6Ag103973) is 7952 bp long and contains 8 exons. This gene would encode a larger protein, 782 aas, with two Glyco hydro 28 domains characteristics of PGs, the first part of the protein (394 aas) identical to FaPG1 protein. If this result is due to a sequencing error or a gene duplication in cv. “Royal Royce” should be determined. FxaC_21g15770 sequence was used to select a sgRNA for inducing single mutations in the gene. The target site of the chosen sgRNA was located 50 bases after the start codon within the first exon, which encodes part of the Glyco hydro 28 domain (Supplementary Fig. 1). To determine any allelic variation within this region that could affect CRISPR/Cas9 editing, *FaPG1* was amplified from cDNA of ripe fruits, cloned into vector pGEMT-easy, and Sanger sequenced. None of the 18 clones sequenced showed polymorphism in the region targeted by the selected sgRNA. Putative *FaPG1* homoeologous were identified in the Royal Royce reference genome (Fxa6Bg103669, Fxa6Cg103555 and Fxa6Dg103454). All of them had a 29 bp deletion close to the target site for the induced mutation that included 9 nucleotides of the sgRNA. Therefore, the selected sgRNA was cloned into the pEn-Chimera vector under the control of the AtU6–26 promoter and later transferred to the binary vector pDe-CAS9, which confers plant resistance to phosphinothricin.

**Figure 1:**
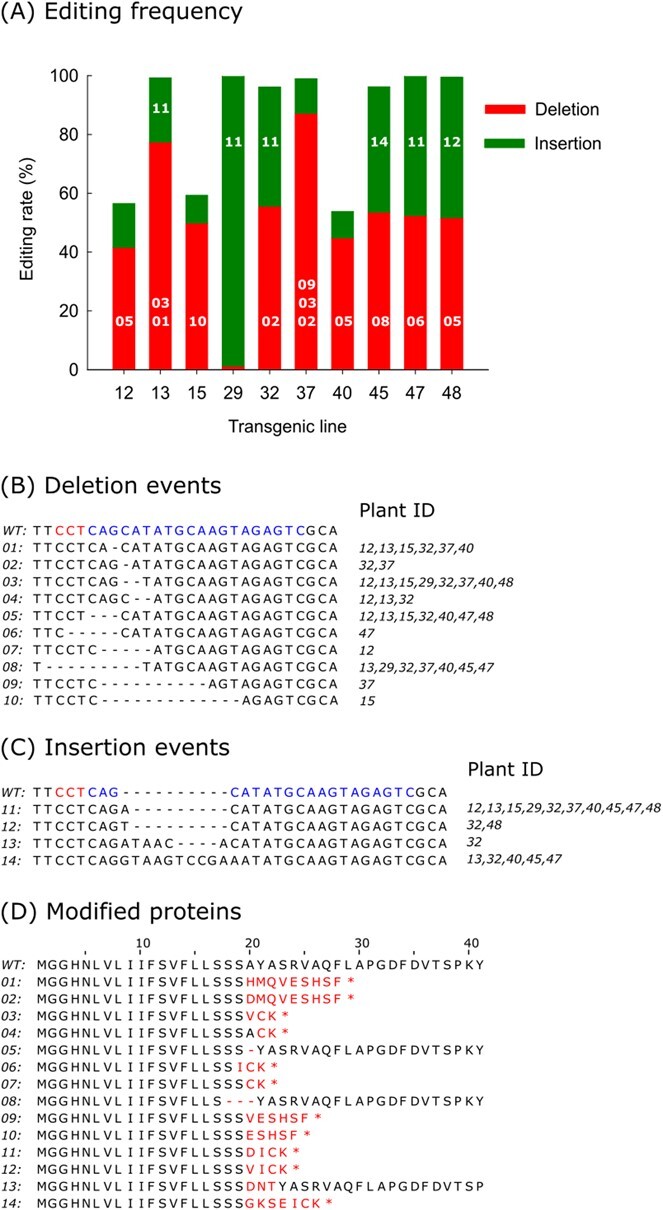
CRISPR/Cas9 editing of *FaPG1* gene in strawberry. (A) Editing rates in the different transgenic lines studied. Numbers inside the bars indicate the main deletion or insertion events found in each line. Deletion (B) and insertion (C) events detected in edited plants. Codes of editing sequences are shown on the left column (from 01 to 14), sequence alignment with wild type sequence (WT) on the central column and plant ID line on the right column. In the wild type sequence (WT), the target sequence (single guide RNA) is indicated in blue and protospacer adjacent motif (PAM) in red colour. (D) Fragment of the translated FaPG1 protein, from 1 to 41 amino acids, containing the CRISPR/Cas9 site of cleavage. Amino acids in red colour indicate transcoding sequences compared to wild type reference. Deleted amino acids and stop codons are indicate by traits and asterisks, respectively. Numbers on the left column correspond to the code of the editing event shown in B and C.

Leaves from micropropagated strawberry plants, cv. “Chandler”, were transformed with *Agrobacterium tumefaciens* harbouring the pDe-CAS9 vector. More than 15 phosphinotricin-resistant lines were recovered, and ten were randomly selected for further experiments. The transgenic nature of these plants was confirmed by amplifying a fragment of 351 bp corresponding to the *bar* gene (Supplementary Fig. 2). The T7 endonuclease I assay was employed to identify primary transgenic lines with the *FaPG1* gene edited. All the plants analyzed displayed a double band in agarose gel when the products of PCR amplification of a fragment of the gene that contained the target sequence were digested with the T7 endonuclease, indicating the successful *FaPG1* editing (Supplementary Fig. 3).

**Table 1:** Characteristics of ripe fruits in wild type and transgenic plants. Data represent means±SD of a minimum of 10 fruits per line. Lines that are significantly different from the wild type by *t*-Student (length, a^*^) or Mann Whitney U (weight, width, SS, L^*^, b^*^) tests with Bonferroni adjustment at *P* = 0.05 are indicated with asterisks

Genotype	Weight (g)	Length (cm)	Width (cm)	SS (°Brix)	Color		
					L^*^	a^*^	b^*^
WT	8.6 ± 2.9	3.3 ± 0.5	2.3 ± 0.3	6.7 ± 1.3	35.4 ± 3.2	40.2 ± 3.9	20.4 ± 4.9
12	7.0 ± 0.9^*^	2.8 ± 0.4^*^	2.3 ± 0.5	5.3 ± 1.2^*^	40.4 ± 4.9^*^	40.4 ± 4.5	26.0 ± 6.7^*^
13	10.1 ± 3.4	3.5 ± 0.6	2.7 ± 0.4^*^	6.2 ± 1.8	33.7 ± 3.0^*^	37.7 ± 3.4^*^	20.0 ± 4.8
15	10.8 ± 3.9	3.1 ± 0.6	2.9 ± 0.5^*^	7.3 ± 2.1	34.1 ± 2.9	40.4 ± 3.4	20.0 ± 5.0
29	6.6 ± 2.2^*^	2.9 ± 0.5^*^	2.2 ± 0.3	6.6 ± 1.2	36.6 ± 4.0	41.3 ± 5.0	22.6 ± 6.0
37	6.0 ± 1.8^*^	2.6 ± 0.5^*^	2.2 ± 0.3	6.8 ± 1.5	37.5 ± 2.3^*^	40.2 ± 4.2	24.5 ± 3.6^*^
40	9.0 ± 3.3	2.8 ± 0.5^*^	2.6 ± 0.4	6.2 ± 1.1	39.1 ± 3.1^*^	41.9 ± 3.4	25.2 ± 4.5^*^
45	6.5 ± 2.3	2.4 ± 0.6^*^	2.2 ± 0.4	6.9 ± 1.3	43.0 ± 4.8^*^	38.9 ± 4.3	27.2 ± 4.8^*^
47	7.0 ± 2.6	2.8 ± 0.5	2.3 ± 0.3	9.2 ± 1.9^*^	37.5 ± 2.9	38.4 ± 5.6	21.1 ± 2.6

### Targeted deep sequencing and bioinformatic analyses

Targeted deep sequencing of genomic DNA was used to determine the mutation efficiency and pattern in the *FaPG1* locus. Between 250 000 and 460 000 sequences per PCR amplification sample were obtained. These sequences were compared to the reference sequence from non-transgenic “Chandler”, and a cutoff of 3000 reads, around 1% of the average value of total reads per sample, was fixed to consider a particular sequence as edited. The percentage of edited sequences in the analyzed fragments varied from 47 to almost 100% (Supplementary Table 1). On average, seven out of the ten lines analyzed showed indel frequencies higher than 96% (lines 13, 29, 32, 37, 45, 47 and 48), while these percentages varied from 54 to 59% in the rest of the lines (lines 12, 15 and 40) (Fig 1A).

An exhaustive analysis of mutation events detected in the cleavage site was performed (Fig. 1B,C). A total of 14 different combinations of edited sequences were identified in the ten plant lines studied. Ten out of the 14 editing events corresponded with deletions (71% of the editing events) and four to insertions (29%). Deletion events number 01, 03, 05, and 08 were the most frequent and appeared in 60–80% of the lines. These events corresponded to the loss of 1, 2, 3 and 9 nucleotides within the cleavage site. By contrast, four editing events, numbers 06, 07, 09 and 10, were detected only in a single transgenic line. As regards insertions, event 11, insertion of a single nucleotide, was detected in all lines, and event number 14, +10, appeared in half of the lines.

The combinations of mutation events detected in each line ranged from 3 in lines #29 and #45, to 7 in lines #12, 13 and 37. Only line #32 displayed a higher number of editing events, 10. However, in all the lines, most edited reads corresponded only to 1 to 3 editing events, while the rest of the mutations represented less than 2% of total reads. The predominant events per line are indicated in Fig. 1A. The mutation pattern was different in most lines, except for lines #12 and #40, which shared deletion event number 05 as predominant.

The deduced amino acid sequences of the FaPG1 protein for all the editing events are shown in Fig. 1D. Eleven sequences showed frame-shifting and premature termination of protein transduction due to the introduction of stop codons. Events 05 and 08 preserved the ORF but caused the loss of one or three amino acids, respectively. Finally, in event number 13, the alanine residue in position 20 was substituted by aspartic acid, and two additional amino acids (asparagine and threonine) were introduced in position 21.

### Phenotypic analyses of transgenic plants

**Figure 2:**
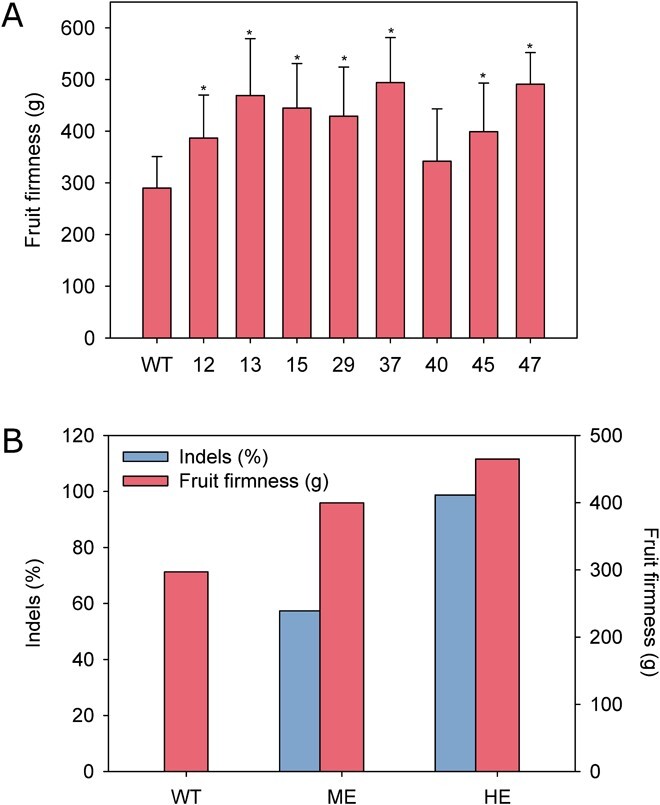
Effect of *FaPG1* mutation in fruit firmness. (A) Firmness of ripe fruits in wild type and edited strawberry plants. Data represent means±SD. Asterisks indicate differences with the wild type by Mann Whitney U test with Bonferroni correction at P = 0.01. (B) Relationship between *FaPG1* editing frequency and fruit firmness. WT: control untransformed plants; ME: average indel frequency and fruit firmness in lines showing indel frequencies lower than 60%; HE: average indel frequency and fruit firmness in lines showing indel frequencies higher than 95%.

Primary transgenic lines were acclimated to greenhouse conditions and propagated by runners. Vegetative growth of edited plants was similar to wild type. Fruits from eight selected lines were harvested at the red ripe stage, with full fruit surface red, and quality parameters were analyzed (Table 1). Fruit fresh weight was significantly lower than wild type in edited lines #12, 29, and 37. Fruit length was also reduced in 5 out of the eight lines evaluated, while the fruit width was not affected in these lines. The ratio length/width was lower than the wild type in most lines, and transgenic fruits were less elongated and squarer when compared to the control. The soluble solids content was similar in control and transgenic fruits except for those from line #12, which displayed a lower value, and those of line #47, higher SS average value. As regards colour parameters, fruits from edited lines #12, 37, 40 and 45 displayed higher lightness (L^*^) and yellowness (b^*^) average values but redness (a^*^) was not modified. By contrast, L^*^ and a^*^ were significantly lower than the control in line #13. Fruit firmness was significantly higher than wild type in all edited lines analyzed except in line 40 (Fig. 2A), which showed the lowest mutation frequency. The increase in firmness ranged from 33% in line #12 to 70% in #37. When grouping the transgenic lines in medium (lines #12, 15 and 40) and high (lines #13, 29, 37, 45 and 47) edited based on the percentage of indel frequencies, the relationship between the degree of *FaPG1* mutation and the increase on fruit firmness was evident (Fig. 2B).

**Figure 3:**
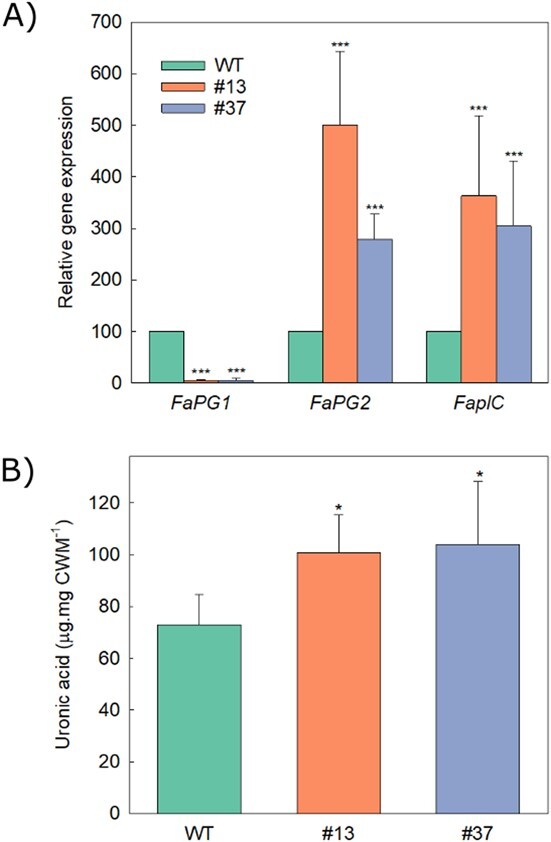
Effect of *FaPG1* mutation in expression of genes involved in pectin degradation and pectin content. (A) *FaPG1*, *FaPG2* and *FaplC* relative gene expression in ripe fruits from edited lines #13 and #37. Data represent mean ± SD of five independent experiments. (B) Uronic acid content in cell wall material (CWM) isolated from ripe fruits of control (WT) and edited lines #13 and #37. Data represent mean ± SD of three independent extractions. Statistical significance with respect to the WT sample was determined by the Student’s t-test: ^*^P = 0.05, ^***^P = 0.001.

Transgenic lines #13 and #37, which showed high fruit firmness and *FaPG1* editing rates, were selected for further studies in the next year, using plants obtained by runner propagation for these experiments. Ripe fruits from line #13 displayed similar values of soluble sugars, pH, titratable acidity and anthocyanin content to the control. Soluble solids and anthocyanin content were significantly higher in fruits from line #37 compared to the control (Supplementary Table 2). Expression of *FaPG1*, *FaPG2* and the pectate lyase gene *FaplC* was also measured in ripe fruits. *FaPG1* was significantly downregulated in both selected edited lines when compared with wild type; by contrast, both *FaPG2* and *FaplC* genes were overexpressed (Fig. 3A). Total amount of pectins in cell wall material (CWM) isolated from ripe fruits was 35% higher in both edited lines than in control (Fig. 3B). Finally, *FaPG1* mutation slightly reduced total PG activity although the differences with control fruits were not significant (0.11 nmol galacturonic acid.μg protein^−1^.h^−1^ in control fruits vs 0.04 and 0.07 nmol galacturonic acid.μg protein^−1^.h^−1^ in edited lines #13 and #37, respectively).

**Figure 4:**
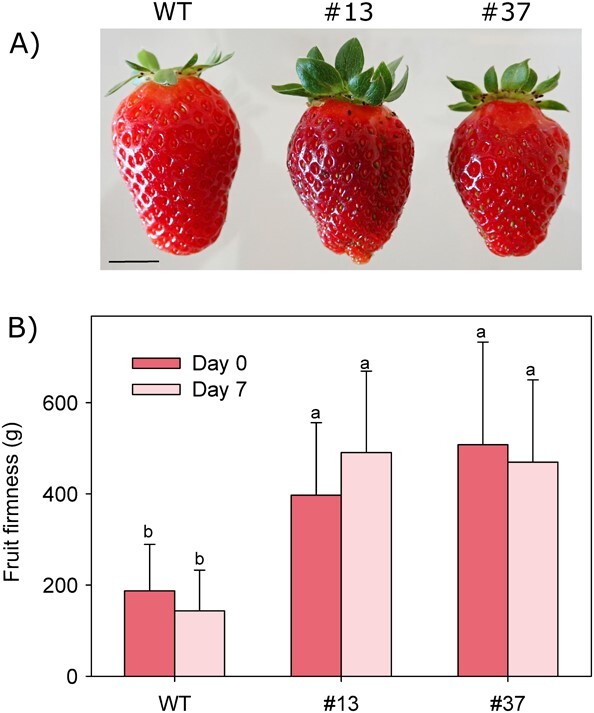
Postharvest study in edited fruits. (A) Aspect of ripe fruits from wild type and transgenic edited lines at harvest. Scale bar corresponds to 1 cm. (B) Fruit firmness before (day 0) and after postharvest storage for 4 days at 4°C followed by 3 days at 25°C (day 7). Data represent means±SD. Bars with different letters indicate significant differences by Tukey test at P = 0.05.

*FaPG1* edited fruits, lines #13 and #37, were also subjected to a postharvest study (Fig. 4). To this purpose, ripe fruits were stored for four days at 4°C, followed by three additional days at 25°C. Fruit firmness was measured before and after the postharvest period using a TA-XT plus texturometer. Mean fruit firmness was not statistically different before and after the postharvest treatment in any line analyzed, mainly due to the large variability in the samples (Fig. 4B). A 27% of wild type fruits acquired a semi-liquid texture after postharvest, with firmness values lower than 50 g; however, none of the edited fruits reached this degree of softening. Even more, the increase in firmness in edited fruits when compared with control was higher after seven days of postharvest in both edited lines than before postharvest (112% vs 242% in line #13, before and after postharvest, respectively; 171% vs 236% in line #37), indicating a lower rate of fruit softening in edited fruits.

**Figure 5:**
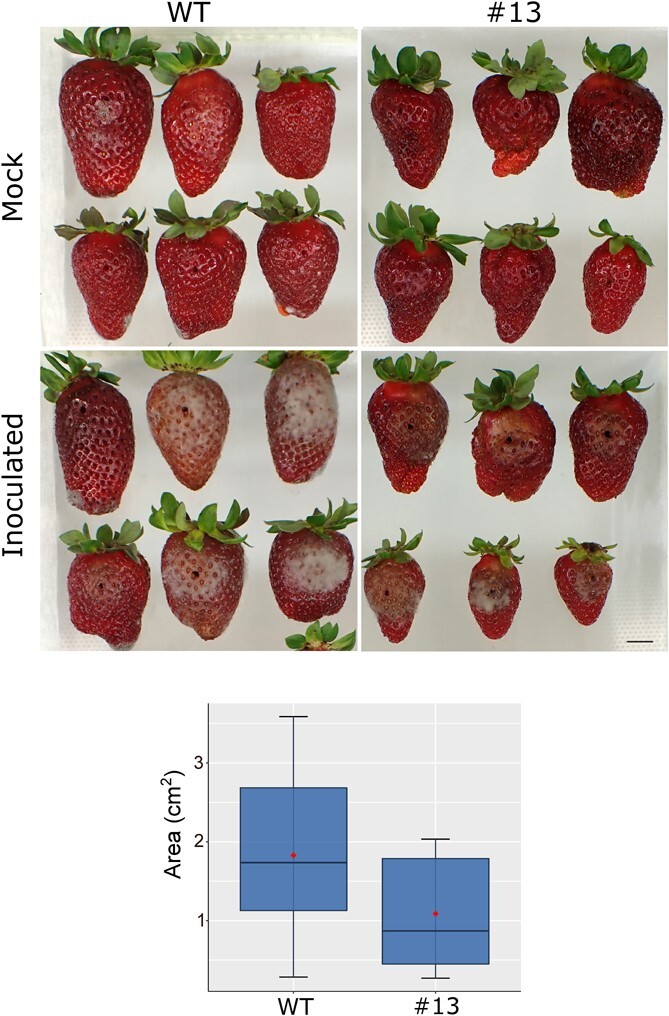
Susceptibility to *Botrytis* in wild type and *FaPG1* edited line #13. Ripe fruits were inoculated by injection of 7 $\mu$l of a *Botrytis* spore suspension at 2 x 10^5^ spores mL^−1^, above the equatorial axis of the fruit, and incubated 4 days at 20°C. A representative picture of mock and *Botrytis* inoculated fruits after the treatment is shown in the upper figure. Scale bar corresponds to 1 cm. The surface showing visible symptoms of infection was also recorded and the box plot is displayed in the lower figure. Mean values of the damaged surface (represented by red points) in WT and line #13 were significantly different by Student *t*-test at P = 0.05

In this postharvest experiment, it was observed that the incidence of fungal rot symptoms after seven days of postharvest was lower in edited fruits when compared with controls. To further investigate the susceptibility of edited fruits to *Botrytis*, ripe fruits from wild type and transgenic line #13 were inoculated with *Botrytis cinerea* strain B05.10 and stored at 20°C for 4 days. No visible damage symptoms were observed in fruits inoculated with distilled water (Fig. 5). The surface with external rot symptoms and/or mycelial growth around the inoculation point was significantly lower in the edited fruits when compared with controls (Fig. 5).

The rate of water loss was also measured in wild-type fruits and edited line #13. To this purpose, fruits were saturated overnight in distilled water and then stored in closed chambers at a constant temperature, 24°C, and relative humidity (RH), 65%. In both genotypes, fruit relative water content (RWC) decreased linearly with time. However, the slope of the curve was lower in edited fruits, indicating a lower transpiration rate (Supplementary Fig. 4). The differences in RWC between control and edited fruits started to be statistically significant after six h of incubation.

### Off-target detection

Putative off-target genes were searched using CRISPOR web tool (http://crispor.tefor.net/) and the *F. vesca* genome. Four genes with off-target scores from 0.61 to 0.02 were identified and analyzed. Orthologous genes in *F.* × *ananassa* (cv. Royal Royce genome v1.0) and their corresponding off-target scores are shown in Supplementary Table 3. Genomic DNA from the five lines with the highest editing percentage (lines #13, 29, 37, 45, 47) was subjected to PCR amplification using specific primers for these genes (Supplementary Table 4). The T7 endonuclease I assay was performed in the amplification products to detect editing events. No off-target mutations in these genes were detected using this assay (Supplementary Fig. 5).

## Discussion

In this study, we have successfully edited the polygalacturonase gene *FaPG1* in cultivated strawberry using the CRISPR/Cas9 system delivered by *A. tumefaciens*. All the evaluated transgenic lines were successfully edited, as detected by the T7 endonuclease I assay and Illumina MiSeq sequencing of amplicons spanning the target site. The editing efficiency ranged from 47 to 100% of the sequenced reads. Similar editing efficiencies have been reported in *Fragaria* and other species. Wilson et al. [18] edited the phytoene desaturase gene in *F. vesca* and *F. × ananassa* cv. “Calypso”. The transgenic lines analyzed showing the expected albino phenotype displayed a broad range of edited reads, from 60 to 100% in both species. Similarly, Pompili et al. [34] edited a gene related to susceptibility to *Erwinia amylovora* in apple; they found that among the edited plants, some were edited entirely, showing single or multiple mutation profiles, while others had a partially edited genotype, being wild-type background maintained.

As expected, most mutation events found in this research corresponded to small base deletions inside the guide RNA, with a lower frequency of insertions. All the edited plants displayed a few mutation events, one to three, that accounted for most of the edited sequences. Wilson et al. [18] described similar results in *F. vesca*, but no insertions or substitutions were reported in *F. × ananassa*. Fungal and plant PGs contain four conserved motifs that play different structural roles, i.e. be components of the catalytic site, participate in the catalytic reaction, and interact ionically with the pectin substrate [35–37]. The analysis of the deduced protein sequences translated from mutation events showed that most of them introduced stop codons within exon 1, resulting in premature termination of protein transduction. By contrast, three of these mutation events preserved ORF but caused the loss or the insertion and substitution of a few amino acids. In these cases, the four conserved motifs typical of PGs were maintained, but the influence of these modifications in protein stability and/or activity is unknown.

Edited lines showed a firmer fruit phenotype due to *FaPG1* knockout. Interestingly, there was a clear positive relationship between the degree of *FaPG1* editing and fruit firmness at harvest. The four lines with the highest editing efficiencies (> 95%) produced fruits that were, on average, 57% firmer than the wild type, being two of the lines almost 70% firmer than the wild type. However, lines with a lower editing rate (c. 57%) showed a 34% increase in fruit firmness. Besides the editing rate, mutation type could also influence fruit firmness. Two lines with low editing rates, #12 and 40, shared the editing event 05 as predominant. This mutation preserved ORF but caused the loss of a single amino acid. These lines also displayed the lowest increase in fruit firmness. Thus, this mutation could have a minor effect on PG activity.

After seven days of postharvest, a significant proportion of control fruits displayed a semi-liquid texture due to over-softening. However, none of the fruits from selected transgenic lines reached such low firmness values. Moreover, the increase in fruit firmness compared with control fruits was even higher after the postharvest treatment, suggesting a slowdown of the softening rate due to *FaPG1* knockout.

Editing *FaPG1* was not only beneficial for reducing the softening rate in strawberry, but also to reduce fruit susceptibility to *Botrytis cinerea* infection and to reduce transpirational water loss. Down-regulation of a PG gene in apple also reduced the loss of water in stored fruits and this was linked to a lower expansion of hypodermal cells, resulting in more densely packed cells in this layer [12]. Apple skin comprises a dense cuticle, an epidermis layer and several layers of hypodermic cells that are swollen during ripening. With this tissue structure, the loss of water and, consequently, cell turgor during apple postharvest occur slowly, lasting almost 20 days to lose 10% of the initial weight [12]. Outer layers of strawberry fruit, by contrast, are composed of a thin cuticle, a layer of epidermal cells and a single second layer of hypodermal cells [38]. This results in significant losses of water in a few days after harvest. The lower transpiration rate of edited *FaPG1* fruits was likely related to differential water relation parameters of cortical cells rather than to a modification of the hypodermis layer. Fruit water potential (Ψ) was close to 0 in wild type and *FaPG1* edited strawberries saturated in distilled water, and decreased at a similar value (around −2 MPa) after three days of desiccation at 65% RH (results not shown); however, the transpirational water loss was significantly reduced in edited fruits. The bulk elastic modulus (ε) represents the ratio of the change in cell turgor to that in the relative cell volume; lower ε values denote more elastic cell walls [39]. An approximation to ε values in strawberry fruit can be deduced from the desiccation experiment performed in this research using the equation ε = (ΔΨ/ΔRWC)*RWC. According to that, *FaPG1* edited fruits displayed higher ε values than wild type; therefore, the lower water loss in *FaPG1* knockout fruits was likely related to more rigid cell walls, as result of a reduced cell wall dismantling. The higher integrity of cortical cell walls and the reduced fruit water loss could also contribute to the lower susceptibility of edited fruits to *Botrytis*. Experiments are in progress to determine if defence pathways are faster and/or strongly activated in knockout *FaPG1* fruits after pathogen inoculation.

Previous studies showed that the silencing of *FaPG1* by genetic transformation with an antisense sequence under the control of the *CaMV35S* promoter reduced *FaPG1* mRNA level to 90–95% in some of the transgenic lines, and this resulted in firmer fruits [14]; however, the increase on firmness was slightly lower than the one achieved in the present research. The antisense silencing of a different PG gene in strawberry, *FaPG2*, also induced an increase in fruit firmness, and, interestingly, there was not an additive effect when both genes were simultaneously down-regulated, suggesting that they could have a redundant role in cell wall disassembly [16]. RNAseq studies of these transgenic fruits showed that PG silencing reduced the expression of other non-related genes encoding cell wall enzymes, remarkably pectin methyl esterases (PME). This result might be explained by the release of oligogalactuoronides (OGAs) by PGs that, in positive feedback, trigger cell wall disassembly [16]. The results obtained in this research reinforce this hypothesis. Although the RNA guide was designed to introduce mutations in the coding sequence of *FaPG1*, transgenic lines displayed a down-regulation of this gene and, by contrast, an up-regulation of *FaPG2* and *FaplC*. The products of these genes have the same target on the cell wall, demethylated pectins. Despite this increase in *FaPG2* and *FaplC* mRNA levels, cell walls from edited fruits contained 35% more pectins than wild type. These results indicate that *FaPG1* have a more significant impact on pectin disassembly during fruit ripening than the other genes. A higher level of pectin methylation due to the down-regulation of PME via OGAs signalling would also prevent the action of *FaPG2* and *FaplC*. Experiments are in progress to characterize cell wall composition in edited fruits. In summary, the results obtained in the present work confirm the key role of *FaPG1* gene in strawberry fruit softening and, for the first time, demonstrate that PG knockout reduces fungal susceptibility and transpirational water loss, globally resulting in improved postharvest shelf life.

On the other hand, unwanted mutations in off-target genomic sites are a potential issue when using CRISPR/Cas9 for plant breeding improvement [40]. The RNA guide designed in the present work was highly precise, and no off-target mutations were detected in any of the plants analyzed that showed the highest *FaPG1* editing rates using the T7 assay. Whole genome sequencing of mutant plants would be needed to confirm this result. Delivery of CRISPR/Cas9 ribonucleoprotein complexes (RNPs) to plant cells through protoplast transfection has been proposed as an alternative approach to reduce off-targeting effects as these complexes are degraded rapidly by endogenous proteases in cells [41]. Additionally, this strategy would facilitate the public acceptance of edited plants since no foreign DNA is integrated into the plant genome. Along this line, a protocol for the isolation and culture of strawberry cv. “Chandler” protoplasts has recently been reported [42], and this is currently being used to set up the editing of the *FaPG1* gene by RNPs.

Attempts to reduce fruit softening by editing genes related to cell wall disassembly has only been reported in tomato [43], a climacteric fruit with a ripening behaviour and textural properties quite different to strawberry. As far as we know, this is the first time CRISPR/Cas9 has been employed to modify strawberry fruit texture, an agronomical trait of great importance in soft fruits. The results obtained suggest that editing genes encoding cell wall pectinases could be an excellent way to improve the fruit shelf life of elite strawberry genotypes.

## Material and methods

### Plant material

Strawberry plants (*Fragaria × ananassa* Duch., cv. “Chandler”) were obtained by runner propagation. Plants were grown in 22 cm diameter pots containing a mixture of peat moss, sand and perlite (6:3:1), and cultured in a confined greenhouse with a cooling system, 30°C maximum temperature, and daylight conditions.

### Design of sgRNAs and construction

The genomic sequence from the gene AF380299.1, annotated as endopolygalacturonase *FaPG1*, was obtained from Redondo-Nevado *et al*. [15]. Single-guide RNA (sgRNA) was designed using the CRISPR-P 2.0 web tool (http://crispr.hzau.edu.cn/CRISPR2/). A sequence of 20 nt CRISPR target sequence upstream of a NGG protospacer adjacent motif (PAM) was selected and blasted against *F. × ananassa* Camarosa v1.0.a2 reference genome (https://www.rosaceae.org/; [32]) in order to ensure the specificity of the sequence.

To determine any polymorphism within the target region of the selected sgRNA, total RNA from a pool of 10–15 ripe strawberry fruits was extracted using CTAB method [44]. cDNA was synthesized using iScript™ cDNA Synthesis Kit (Bio-Rad Laboratories, Inc.) and cloned into pGEMT-Easy vector system (Promega, Madison, USA). Positive colonies were sequenced by Sanger, and the sequences were aligned using Multiple Sequence Comparison by Log-Expectation (MUSCLE) with Snapgene (https://www.snapgene.com/).

Plasmids pEn-Chimera and pDe-CAS9 used for *FaPG1*:pDeCas9 construct were provided by Botanisches Institut (www.botanik.kit.edu/crispr). Assembly of sgRNA in pEn-Chimera vector was performed according to the protocol described by Schmil *et al.* [45]. First, sgRNA was cloned into pEn-Chimera vector previously digested with *Bbs*I and purified. *E.coli* TOP10 strain was transformed and plated on LB medium supplemented with ampicillin. Single colonies were checked by colony-PCR, and three clones were sequenced with SS42 primer (Supplementary Table 4) to verify the correct integration of spacer. Then, sgRNA was transferred by Gateway reaction to pDe-CAS9 vector; this vector contains the *bar* gene for plant phosphinothricin (PPT) resistance. *E.coli* TOP10 strain was transformed with this vector and plated on LB supplemented with spectinomycin. Single colonies were checked by PCR and the purified plasmid was sequenced using primers SS42 and SS43 (Supplementary Table 4). The final construction was introduced in *A. tumefaciens,* AGL1 strain, for plant transformation.

### Generation of transgenic strawberry plants

*Agrobacterium*-mediated transformation experiments were performed as previously described [16, 46], using leaf disks from micropropagated plants as explants [16, 46]. The micropropagation medium contained N30K [47] macroelements, MS [48] microelements and vitamins, and 2.21 μM kinetin. Leaf disks were incubated for 8 days in shoot regeneration medium, a modified MS medium with N30K macroelements, 8.88 μM benzylaminopurine (BA) and 2.46 μM indole-3-butyric acid (IBA). Then, explants were inoculated with *A. tumefaciens* strain AGL1 carrying the construct FaPG1:pDeCas9, diluted to a OD_600_ = 0.2. Explants were colcultured for 2 days and later transferred to selection medium, i.e. regeneration medium supplemented with 5 mg l^−1^ PPT and 250 mgl^−1^ timentin. Regenerated shoots were micropropagated and rooted in the presence of 5 mg l^−1^ PPT, and acclimated to ex vitro conditions. Primary transgenic plants were propagated by runners in the greenhouse, and daughter plants were used for phenotypic analysis.

### DNA extraction

Young strawberry leaves were used for genomic DNA extraction. After powdered in liquid nitrogen, plant material was washed three times in washing buffer (100 mM sodium acetate buffer, pH 5, 20 mM EDTA, 0.2 M sorbitol, 2% polyvinylpyrrolidone (PVP, molecular weight 40 000), and 1% β-mercaptoethanol). Then, DNA was extracted using the CTAB method [49]. Quality was checked by quantification in nanodrop (Thermo Sci) and resolved in 0.8% agarose gel.

### Selection of transgenic and CRISPR/Cas edited plants

The transgenic nature of PPT resistant plants was confirmed by PCR amplification using specific primers for the *bar* gene (Supplementary Table 4). Edited plants from transgenic lines were selected by performing the T7 endonuclease I assay that recognizes and cleaves non-perfectly matched DNA [50]. Using flanking primers to sgRNA sequence (Supplementary Table 4) and Phusion High-Fidelity DNA polymerase (NEB), a fragment of 351 bp was amplified by PCR, purified and digested with T7 endonuclease I (NEB) following commercial instructions. Digested PCR products were resolved in 1.5% agarose gel. Those lines with a double band of around 350 bp were selected as edited lines.

### Targeted deep sequencing

Three biological replicates of gDNA from 10 independent edited lines and wild type, non-transformed plants were sent for high-throughput sequencing using Illumina MiSeq platform. First, DNA quality was checked and then amplified using the previously mentioned flanking primers (Flanq_Guia67_F and Flanq_Guia67_R) with Illumina adaptors. Finally, paired-end sequencing (2x250 cycles) was performed using this technology.

The results obtained from the high-throughput sequencing run were analyzed using the Cas-Analyzer tool from CRISPR RGEN software (http://rgenome.net/). Raw paired-ends reads files were uploaded, and the sequence of the amplified fragment of 351 bp of “Chandler” cultivar sequence obtained from the database was fixed as the reference sequence. Parameters such as paired nuclease, Cas9 nickases, SpCas9 from *Streptococcus pyogenes*: 5´-NGG-3, comparison range of 70 nt and 3000 reads as minimum frequency were selected.

### Off-target detection

Putative off-targets were identified using CRISPOR web tool (http://crispor.tefor.net/) and the *F. vesca* genome v4.0.a1. Homologous genes in *F.* × *ananassa* were searched using the cv. Royal Royce genome v1.0 (https://www.rosaceae.org/). Genomic DNA from control and transformed plants was extracted following the protocol previously described. DNA fragments containing the off-target site were amplified by PCR using the primers indicated on the CRISPOR website (Supplementary Table 4). PCR products were purified, treated for T7 endonuclease I and resolved in 1.5% agarose gel.

For each putative off-target sequence analyzed, gDNA from non-transformed plants was included as a negative control. The amplification of the fragment containing sgRNA for *FaPG1* was included as a positive control.

### Phenotypic analyses of transgenic plants

Transgenic plants grown in a confined greenhouse, as previously described, were evaluated during the growing seasons of 2020 to 2022, using non-transformed plants as control. Each year, control and edited lines were propagated by runners and the daughter plants were used for evaluation. Fruits were collected from March to July at the stage of full ripeness (approximately 25 to 30 days after anthesis, with 100% surface red). Six to 15 plants per line were cultivated, and about 10–50 fruits per line were evaluated.

Fresh weight, size, colour, firmness and soluble solids content were recorded in freshly harvested fruits. The colour was measured using a colourimeter (Minolta Chroma Metre CR-400, Osaka, Japan) and the L^*^ a^*^ b^*^ colour space parameters. Firmness was evaluated using a hand penetrometer (Effegi) with a cylindrical needle of 9.62 mm^2^ area. Finally, soluble solids content was measured using a refractometer (Atago N1).

For pH and titratable acidity determination, 5 to 10 frozen fruits were pooled, powdered in liquid nitrogen and 1 g was homogenized in 10 ml of distilled water. After filtration, samples were diluted to 50 ml, and the pH of the solution was recorded. Titratable acidity was measured as described by Nunes et al. [51], and the results expressed as percentage of citric acid. To estimate anthocyanin content, 1 g of fruit was homogenized in 10 ml of methanol:HCl (99:1), and the samples were incubated at 4°C for 1 h. Then, extracts were centrifuged, and the absorbance at 515 nm of the supernatant was measured. Anthocyanin content was expressed as mg pelargonidin-3-glucoside.100 g^−1^ of fruit [52].

For postharvest assays, ripe fruits were stored during 4 days at 4°C followed by 3 additional days at 25°C. Fruit firmness was analyzed in freshly harvested fruits (day 0) and after postharvest (day 7) with a texturometer (Texture Analyser TA-XT plus) using a penetration test with a 6 mm diameter cylinder probe under the next parameters: speed test 1 mm/s; target mode Distance; distance 6 mm; and trigger force 5 g. The maximum force (N) was used to compare firmness in 15–25 fruits per line at each sampling day.

A test for *Botrytis* susceptibility in control and edited ripe fruits was also performed. An insolate of *Botrytis cinerea* strain B05.10, kindly provided by Dr. Vela-Corcia (IHSM “La Mayora”-UMA-CSIC), was grown on PDA medium at 20°C for 14 days under natural daylight conditions for spore production. For inoculum production, *Botrytis cinerea* spores were recovered by flooding the cultures with 5 ml of sterile saline serum, scratching the surface with a 1 mL tip pipette by repeat pipetting. The spore suspension was filtered through two layers of sterile miracloth to disregard mycelia. Spores were counted with a Neubauer chamber and diluted in sterile distilled water to a final concentration of 2 x 10^5^ spores mL^−1^. Ripe fruits were hand-picked from the greenhouse and brought to the laboratory to be used the same day. Only fruits at the full ripe stage and without defects were selected. All fruits were surface sterilized with 10% sodium hypochlorite and 0.1% tween 20 for 2 min followed by three washes with distilled water. Botrytis experiment was done by injection of 7 $\mu$l of spore suspension at 2 x 10^5^ spores mL^−1^ above the equatorial axis of the fruit. Control fruits followed the same treatment, and they were inoculated with 7 $\mu$l of distilled water. Fruits were incubated at 20°C in transparent plastic boxes at high humidity and natural daylight (14-h photoperiod). After 4 days of incubation, the fruits were photographed, and the surface area showing visible symptoms of infection, rot and/or mycelial growth around the puncture point was recorded using the ImageJ software. The experiment was performed in triplicate with at least 8 fruits per replicate.

To determine transpirational water loss, ripe fruits were harvested with a 2–4 cm cut pedicel, transported to the lab and stored overnight in a closed chamber with the pedicel immersed in distilled water. Saturated fruit weight (SW) was recorded after removing the pedicel and sepals. Later on, the fruits were stored in a closed chamber containing a saturated solution of sodium acetate that provides a constant RH of around 65% at 25°C. Fruit fresh weight (FW) was regularly recorded during 3 days. At the end of the experiment, fruits were dried in an oven at 80°C for 3 days to determine dry weight (DW). Relative water content (%) was calculated as 1-((SW-FW)/(SW-DW)). The average temperature and RH inside the chamber during the experiment was 24.2 ± 0.7°C and 63.5 ± 2.9%, respectively. Ten fruits per line were measured, and the experiment was repeated twice.

### Gene expression

Total RNA from control and edited *FaPG1* red fruits was isolated with the Maxwell® 16 LEV Plant RNA kit (Promega, Madison, WI, USA), according to the instructions provided by the manufacturer. The RNA obtained was quantified in a nanodrop 1000 Spectrophotometer (Thermo Fischer Scientific, Waltham, MA, USA), and its integrity was checked using an Agilent 2100 Bioanalyzer (Agilent Technologies, Santa Clara, CA, USA). Reverse transcription was carried out using 250 ng of purified total RNA from each sample with a RIN value of 8 with iScript cDNA Synthesis kit (Bio-Rad, Hercules, CA, USA). qRTPCR was performed using specific primers (Supplementary Table 4) and SsoAdvancedTM SYBR® Green Supermix in a CFX real-time PCR system (Bio-Rad, Hercules, CA, USA). Relative expression values were obtained with the ΔΔCt method [53], using interspacer 26S–18S as housekeeping gene.

### Cell wall extraction and PG activity

Cell wall material (CWM) was extracted from ripe fruits as previously described [54]. Ten g of fruit were extracted with 20 ml PAW (phenol:acetic acid:water), centrifuged at 4000 *g*, and the pellet de-starched with DMSO 90%. Total amount of pectin in the CWM was determined by the carbazole method [55] after hydrolysis with concentrated sulfuric acid.

For PG activity quantification, ripe fruits were homogenized in 50 mM NaOAc buffer, pH 6, containing 1% (w/v) PVPP, centrifuged at 12000 *g*, and the pellets extracted with the extraction buffer containing 1 M NaCl [17]. Then, protein extracts were stirred for 2 h at 4°C, centrifuged at 12000 *g* and the supernatants were dialyzed against 50 mM NaOAc buffer. The reaction mixture for PG activity contained 50 mM NaOAc, pH 5.5, 0.2% polygalacturonic acid and an appropriate volume of protein extract. Reaction mixtures were incubated at 37°C for 24 h, and the reducing groups were measured by the cyanoacetamide method, using galacturonic acid as standard [56]. The protein content of extracts was estimated by the Bradford assay. Three independent extractions per line were performed.

### Statistical analysis

Data were subjected to ANOVA or pairwise comparisons using R. Bartlett test for homogeneity of variance was previously performed. t-Student or Mann Whitney U tests were used for pairwise comparisons in the case of homogeneous and non-homogeneous variances, respectively. Tukey HSD test was used for multiple comparisons. All tests were performed at P = 0.05.

## Acknowledgements

This work was supported by the Ministerio de Ciencia, Innovación y Universidades and FEDER EU funds (grant numbers AGL2017-86531-C2-1R and PID2020-118468RB-C21), and the University of Malaga (grant number B1-2020_09). CS-R was awarded a PhD Fellowship from the Ministerio de Ciencia, Innovación y Universidades (PRE2018-085509), PhD Program Advanced Biotechnology, University of Málaga. We thank Botanisches Institut (Germany) for sharing CRISPR/Cas plasmids for gene editing and Dr. Vela-Corcia (IHSM “La Mayora”-UMA-CSIC, Spain) for providing *Botrytis* strain B05.10.

## Authors’ Contributions

AJM, JM-B and JAM conceived the original project; GL-C, CS-R, CP and PDR-V performed the experiments; SP and RB-P supervised the experiments; GL-C and JAM wrote the article with the contributions of all the authors.

## Data availability

The datasets generated for this study are available on request to the corresponding author.

## Conflict of interests statement

The authors declare that there are no conflict of interest.

## Supplementary data

Supplementary data is available at Horticulture Research online.

## Supplementary Material

Web_Material_uhad011
