# Supplementary material for: CRISPR/Cas9 editing of the polygalacturonase FaPG1 gene improves strawberry fruit firmness
Source: Hortic Res. 2023 Feb 1;10(3):uhad011. doi: 10.1093/hr/uhad011 (PMC10028403; doi:10.1093/hr/uhad011)
Supplement: Web_Material_uhad011 [file web_material_uhad011.zip › Supplementary material-revised_3.pdf]

CRISPR/Cas9 editing of the polygalacturonase *FaPG1* gene improves strawberry fruit firmness

Gloria López-Casado, Cristina Sánchez-Raya, Pablo D. Ric-Varas, Candelas Paniagua, Rosario Blanco-Portales, Juan Muñoz-Blanco, Sara Pose, Antonio J. Matas, Jose A. Mercado

Supplementary Fig. 1: Genomic DNA of AF380299.1 *Fragaria* × *ananassa*, cultivar ‘Chandler’, putative endo-polygalacturonase gene (*FaPG1*). The red colour indicates the oligos for sequencing by Illumina (amplicon size was 351 bp); blue indicates the sgRNA sequence; pink indicates the PAM motif (single guide RNA was designed in the antisense strain). Start and stop codons are shown in bold format.

>AF380299.1

CTGTTATTGAATTTCTATAAAATGTTATAATATTGATTTCTTAATGATCAGTTAACTACGTGATTATTTG
ATATGTTTTTAATCTAAAATGTGATATGTAAAATATAGAAGAAAAAAAATTAAAAAGAACTTTAAGAAAA
AAATTTCAACCCACCCCAACCTAAAATCCTAGGTCCGCCATGGTAATTATAGATATATGATGATGAAGGG
CAAATATTGGTCTATGAGAATTTCGGTGATACTACCGCTTGAAGAGCAATAATGGTTTTGGGACTCCGAT
GAGGGAAACATTCAAATATGATGGATTTTGGTGATACTATGTTTACCCGAGCTAGCTATCACAGAATAAT
CTACATCCCACAAATGAAATATGTTATAGGCTACCAATTAGGAAGTAGTGGAATTATGAAGAAGTAGGGA
TGTGCAAATATAAGAGAAAATTTGAAAATTATGATTGAAACAAGTTATGTTTTTTTAACTAGATGAATTA
AATGGTTTAAAGATTTGTAGATTTATAATCAAACAATTACCGCTACTCTATCGGTGACTACCAATTCCAT
CATTGTAAATAACAAATAACAGATTCGTTGCTGGATGTCTTAGTGCCGTGAAGCCTACAAATCACACTAT
AAACTGCTTAGCTCTCGAGCGTTACTAATTTGGTGATTACCAATTCCAACATTGCGACTTCTTCTACTAG
TAGTACTAAAATAGCAAGTAATATGCATTTGTGGTAAGATGTTTGGTGTTAACCTTTCCTAACCAGACTA
TAAATGACCTCAACACTATAGTGGAGTTTCATCGATCATCATTCTAAACGAAAAACTTGAAGTGAAAGCA
TCAAG**ATG**GGAGGACACAATCTAGTCTTGATTATCTTTTCGGTATTTTTGTTATCTTCCTCAGCATATGC
AAGTAGAGTCGCACAGTTTTTAGCTCCTGGTGACTTTGATGTGACAAGTCCGAAATATGGTGGGAAGCCT
AATACTGATATTAGTCAGGTAAACTATACATATTATAATCGTACAAATACTTCAAAATAGAATGGTTGAT
TTCATTTTTCTTTTTCTTTGTACATAGCCTTTGGCAAATGCTTGGAAAGATGCTTGTGCAGCAACAACGC
CAAGTAGAGTTATTGTTCCAAAAGGGACATTTCAACTAAAAGGAGCAGTTTTCAAAGGTCCTTGTAAGGC
TCCTATTACGGTTCAGGTTGATGGCATACTGCAGGCGCCACCAATTGAAGCCCAATTAGCAAATAAAGAA
TTTTGGGTCCAGTTTTTAGAGGTTGAGAGGCTCACTGTATCAGGTACTGGAACATTTGATGGTCAAGGAC
AAAATTCTTGGAAAGACAACGACTGCAATAAAAATCCAAACTGTGGAGGTCTAGCCATTGTAAGCTAATA
TAGCCCTCTTCTCTGTTTATTTATTTATTTATTTTTTGATGTATTGCATTTCGTGCTAATTTGTTTCAGG
TAACCCTGATTTACATTTTCAAGATATATTGATATATATATATATGTCGTGCTGCTTTCTCATTATGTGC
AGAATGTGAGATTCGACAGAGTGAAAAATTCGTTAGTAAGGGATGTGACATCACTTAACAGCAAAAATTT
CCACTTCAATATTTTAGGGTGTGAACATCTTACATTCCAACATGTCATCGTCAAAGCACCGGGAGATAGC
CCTAACACAGATGGAATACATATGGGGCGCTCAACCAGGATTAACATTACGGACACGAACATCGGAACCG
GAGACGATTGCATATCAGTTGGTGATGGTACCAGACAACTAACTGTAAGTAAGGTAAGTTGTGGACCAGG
TCATGGAATAAGTATCGGAAGTCTCGGAAGATACGACAATGAAGATGACGTCAGTGGACTCAATATTAGA
GATTGTACCCTGAGTAATACACTGAATGGTGTTAGAATCAAGACATTTCCTGCTTCTCCTAAAGCTACCA
CCGCCTCGGATATTCACTTCGAGAAAATCACAATGAATAATGTTGCTAACCCAGTCCTCATTGACCAAGA
ATACTGCCCATGGGGTCAGTGTAATAAACAGGTAATCATCCATGCATCTTAATTACTAACCTACATAATA
CTTCTTTTTCGCGTCTTGGTTACCGATGTACCTCATGATATATGTAGTCTATCAGGTTTCTTAATTAAAT
TCCATTATCCATGATCTTATTAATTATACACAAATCTAATTAGTGATGTTTCTTGCTCGAAGATTCCATC
AAAAGTCAAGATCAGCAATGTGAGCTTCAAGAACATTATAGGGACAACTTCAACTGCGGAGGCTTTGAAG
ATTGTATGCGCTAAAGGCTTGCATTGTGATCAAGTGGTACTGAGTGACATAGATCTCAAGTTAAGTGGCA
AAGGAACTCTTACATCTCATTGTGCAAACGTGCAACCCACGATTACTCGAGTGCCACCGCCTCTTGCTTG
TGCTACCAAGGCTT**TGA**TCATATTGATTAGCAGCTAGAAATAATATGGATCTAAAAGACTTTAGGTGTCAC
GTC

Supplementary Fig. 2: PCR amplification of a 351 bp fragment from *bar* gene for phosphinothricin resistance in DNA isolated from edited strawberry plants. CR-PG1: CRISPR/Cas9 edited lines, each lane corresponds to an independent line; WT: DNA from wild type plants; P: pDeCas9 plasmid.

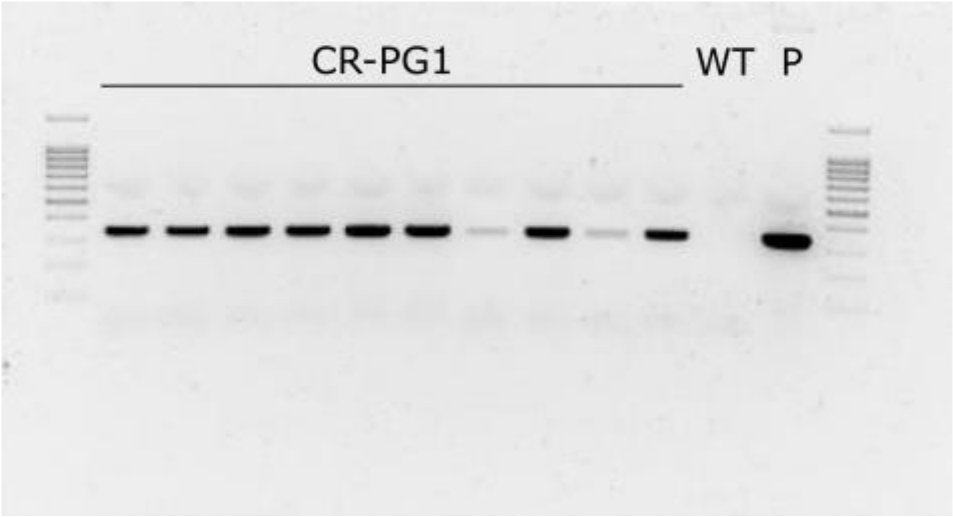

Supplementary Fig. 3: T7 endonuclease I assay in DNA samples from transgenic lines. A fragment of the *FaPG1* gene was amplified by PCR, digested with T7 endonuclease and resolved in a 1% agarose gel. Those lanes showing a double band indicate the successful edition of the gene. CR-PG1: CRISPR/Cas9 transgenic lines, each lane corresponds to an independent line; WT: DNA from wild type plants.

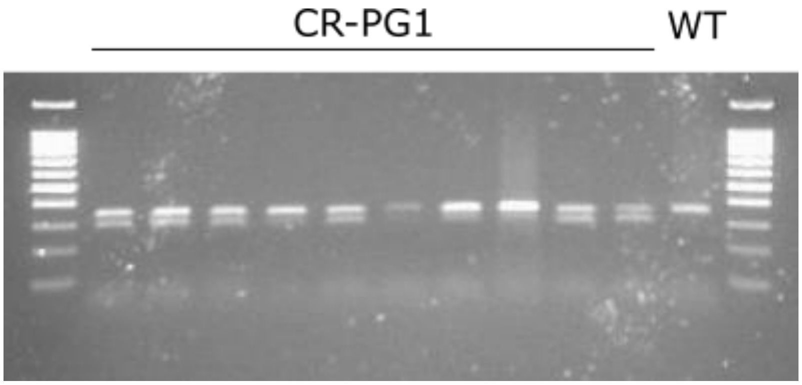

Supplementary Fig. 4. Transpirational water loss in ripe fruits from wild type and FaPG1 edited line #13. Fruits were saturated overnight in distilled water and later stored at 24ºC and 64% RH. The fruit fresh weight was measured at regular intervals during three days. Asterisks indicate significant differences by Student t-test at P=0.05.

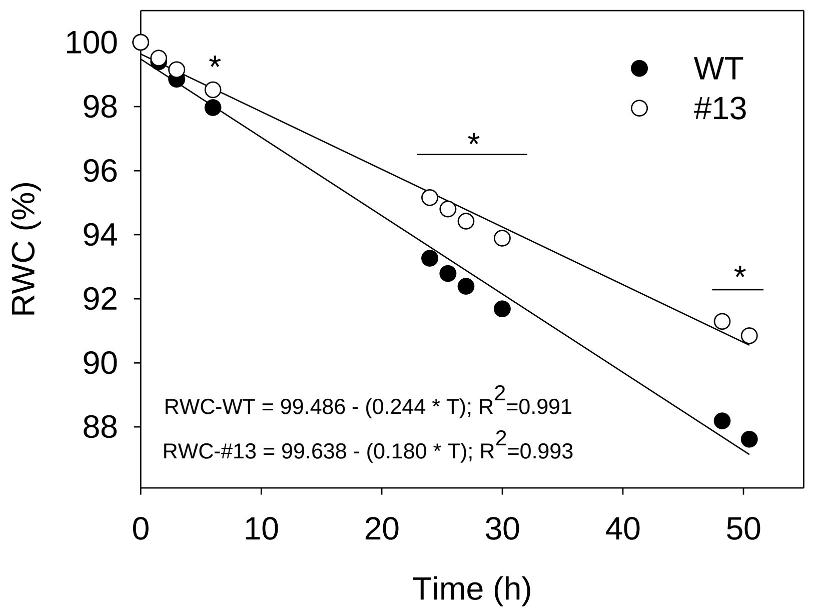

Supplementary Fig. 5. Off-target evaluation in the five lines with the highest editing percentage (Lines 13, 29, 37, 45 and 47). Positive control of the assay (C+) corresponds to T7 endonuclease I performed with one of the selected lines using specific primers for *FaPG1*; negative control (C-) corresponds to wild type plant of ‘Chandler’ cultivar. Each gel (from A to D) corresponds to a different gene identified as possible off-target for this construction.

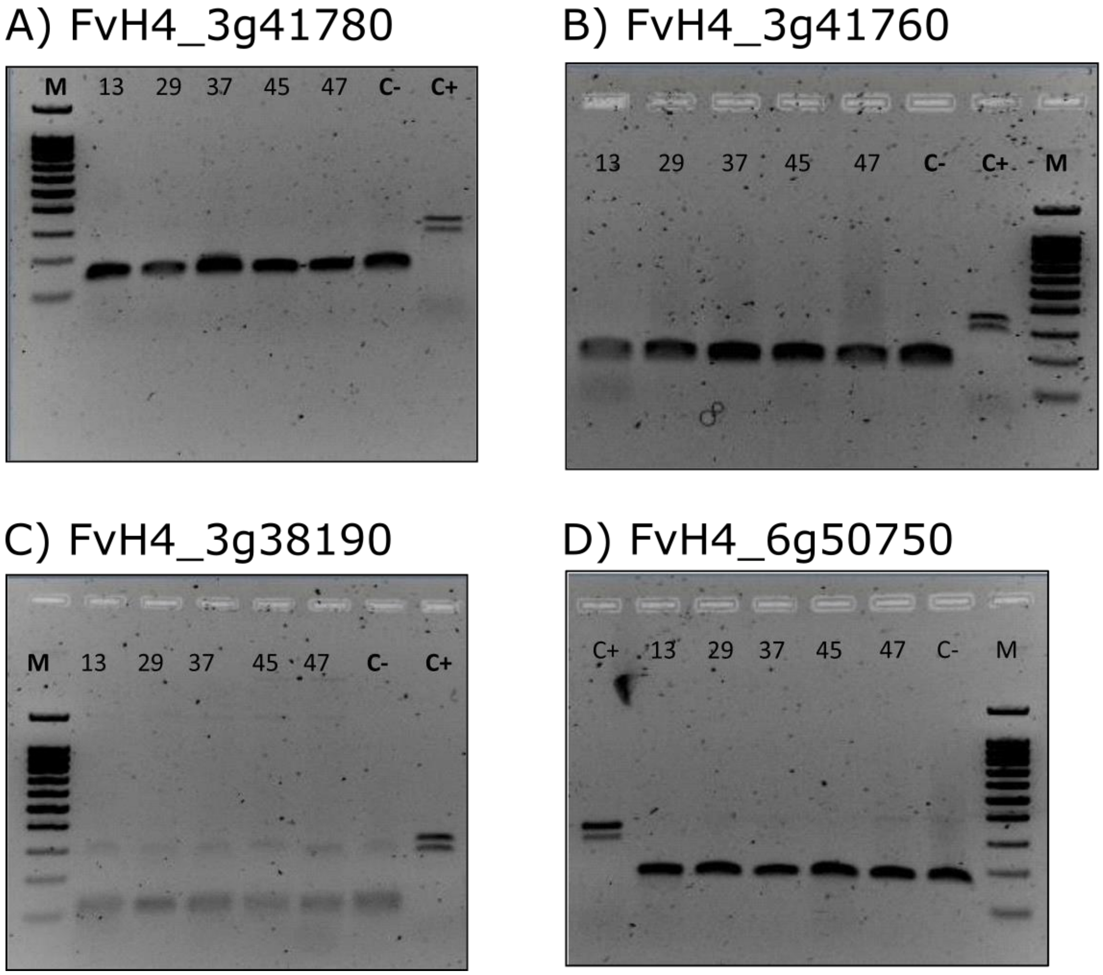

Supplementary Table 1: Summary of sequence analysis using Crispr Rgen Tools software**.** The number of total sequences, wild type and indel sequences are shown. More than minimum frequency indicates that at least there are 3000 counts in the sample with the same modification. Data were analyzed in three biological replicates of 10 independent transgenic lines and 'Chandler' control line.

**REPLICATE 1**											
**Plant Line Code**	#12	#13	#15	#29	#32	#37	#40	#45	#47	#48	Chandler
**Total Sequences**	442074	358198	360976	297906	275011	369080	320282	470703	364167	353101	398795
**With Both Indicators**	429082	338872	326567	272268	249624	342817	299208	460214	340907	331925	363139
**More than minimum**	384716	301021	284709	246903	211819	301021	266427	403220	301596	296361	327881
**Count Type WT**	203951	0	137625	0	0	2164	140124	0	721	0	321620
**Indel Frequency**	180765	303241	147084	246903	211819	301021	126303	403220	301596	296361	6261
**Indel Frequency Percentage**	47.0%	100%	51.7%	100%	100%	100%	47.4%	100%	99.8%	100%	1.9%
**REPLICATE 2**											
**Plant Line Code**	#12	#13	#15	#29	#32	#37	#40	#45	#47	#48	Chandler
**Total Sequences**	309346	399139	302261	369820	290812	383347	381698	210479	347130	422880	371579
**With Both Indicators**	300745	375030	283946	333318	275128	357117	354995	194739	325614	397807	353909
**More than minimum**	269387	327983	248238	297451	244621	316325	319146	167242	286339	356351	320699
**Count Type WT**	136193	0	100727	0	16598	793	123066	17005	1383	5789	303747
**Indel Frequency**	133194	335103	147511	297451	228023	316325	196080	150237	286339	350562	16952
**Indel Frequency Percentage**	49.4%	100%	59.4%	100%	93.2%	100%	61.4%	89.8%	99.3%	98.4%	5.3%
**REPLICATE 3**											
**Plant Line Code**	#12	#13	#15	#29	#32	#37	#40	#45	#47	#48	Chandler
**Total Sequences**	259588	268759	470377	286230	400846	460979	393180	324824	307761	464337	261587
**With Both Indicators**	243438	255478	439601	259855	369521	435875	367744	296253	289342	438621	297260
**More than minimum**	211581	221980	375535	228115	322498	377822	326314	252548	251249	391682	269388
**Count Type WT**	53827	5094	124587	0	15419	11754	154630	0	0	0	261587
**Indel Frequency**	157754	216886	250948	228115	307079	366068	171684	252548	251249	391682	261587
**Indel Frequency Percentage**	74.5%	97.7%	66.8%	100%	95.2%	96.9%	52.6%	100%	100%	100%	0%

Supplementary Table 2: Soluble solids, pH, acidity and anthocyanin content in ripe fruits from wild type and selected edited lines #13 and #37. Data represent mean±SD of at least 24 fruits for SS and three independent extractions for pH, acidity and anthocyanin content. Means with different letters indicate significant differences by Tukey test at P=0.05.

	WT	#13	#37
Soluble solids (ºBrix)	6.8±1.3b	7.2±1.7b	8.4±1.6a
pH	3.15±0.25ab	3.03±0.15b	3.40±0.15a
Titratable acidity (% citric acid)	0.84±0.17a	0.94±0.13a	0.84±0.03a
Anthocyanin content (mg/100g of fruit)	67.4±7.7a	82.1±12.9ab	99.3±7.4b

Supplementary Table 3: List of putative off-target genes analyzed in the edited plants. Off-target genes were identified using CRISPOR web tool (https://www.rosaceae.org/) and the *F. vesca* genome v4.0.a1. Homologous genes in *F.* × *ananassa* cv. Royal Royce reference genome v1.0 are also shown. Off-target scores correspond to the CFD (cutting frequency determination) values estimated by using CRISPOR web tool. CFD ranges from 1 (on-target) to 0.

F. vesca	*F.* × *ananassa*	Off-target score
FvH4_3g41780	Fxa3Ag103907	0.61
FvH4_3g41760	Fxa3Ag103905	0.27
FvH4_3g38190	Fxa3Ag103547	0.09
FvH4_6g50750	Fxa6Ag104843	0.02

Supplementary Table 4: List of oligonucleotides used in this research

**Name**	**Sequence 5'-3'**	**Purpose**	**Reference**
F_Guia67_PG1	ATTGGACTCTACTTGCATATGCTG	Cloning of sgRNA	This research
R_Guia67_PG1	AAACCAGCATATGCAAGTAGAGTC	Cloning of sgRNA	This research
M13rev	CACAGGAAACAGCTATGAC	Colony-PCR	Schiml *et al*., 2016
SS42	TCCCAGGATTAGAATGATTAGG	Plasmid sequencing and Colony-PCR	Schiml *et al*., 2016
SS43	CGACTAAGGGTTTCTTATATGC	Colony-PCR	Schiml *et al*., 2016
BAR-F	CATCGAGACAAGCACGGTCAACTTC	Checking PPT resistance	This research
BAR-R	ATATCCGAGCGCCTCGTGCATGCG	Checking PPT resistance	This research
Flanq_Guia67_F	GAGGACACAATCTAGTCTTG	Flanking primers for amplicon sequencing by Illumina MySeq	This research
Flanq_Guia67_R	CGTAATAGGAGCCTTACAAG	Flanking primers for amplicon sequencing by Illumina MySeq	This research
FvH4_3g41780_F	TGCCGTGAACAGTGTTGACT	Off/targets evaluation	This research
FvH4_3g41780_R	TGCCTTCTGGTGAATGATTTTGG	Off/targets evaluation	This research
FvH4_3g41760_F	CCATGTAGGGCTGCCATGAA	Off/targets evaluation	This research
FvH4_3g41760_R	TGCCTTGTGTGGATGAGTGT	Off/targets evaluation	This research
FvH4_3g38190_F	ATCTTGCAGGTGGGTCTTCC	Off/targets evaluation	This research
FvH4_3g38190_R	AGGGTACTTCAGCTTCCCCA	Off/targets evaluation	This research
FvH4_6g50750_F	CGGAGGATTTGTTGTGGTCG	Off/targets evaluation	This research
FvH4_6g50750_R	ACCCTGACCGGAGATCTTAT	Off/targets evaluation	This research
FaPG1_F	GCTCCTGGTGACTTTGATGT	qRTPCR of *FaPG1* gene	This research
FaPG1_R	ACTCTACTTGGCGTTGTTGC	qRTPCR of *FaPG1* gene	This research
FaPG2_F	GGGCATTCTGGATGACTGGT	qRTPCR of *FaPG2* gene	This research
FaPG2_R	TCCCGGTAAATGGATGGTCG	qRTPCR of *FaPG2* gene	This research
FaplC_F	TGGGTTGATCACAACTCGCT	qRTPCR of *FaplC* gene	This research
FaplC_R	TGCATCTGCTTGTCCCTTGT	qRTPCR of *FaplC* gene	This research
26S-18S_F	ACCGTTGATTCGCACAATTGGTCATCG	Housekeeping gene for qRTPCR	This research
26S-18S_R	TACTGCGGGTCGGCAATCGGACG	Housekeeping gene for qRTPCR	This research
